# A novel maternal prenatal risk index to predict mortality-weighted severe maternal morbidity at hospitalization: a retrospective cohort study

**DOI:** 10.1016/j.lana.2026.101481

**Published:** 2026-04-24

**Authors:** Deborah Kilday, Drew Tatum, Michael Korvink, Kathy W. Belk, Joseph Beals, Ashley Finke, Stephanie Martin, Raymond Perigard, Richelle Marshall, Yue Jiang, Joseph G. Ibrahim, John Yeh, Dorothy A. Fink

**Affiliations:** a U.S. Department of Health & Human Services, Office of the Assistant Secretary for Health, Office on Women’s Health, Rockville, MD, USA; b Advisory Services, Premier Inc., Charlotte, NC, USA; c Institute for Health and Humanity, Medical College of Wisconsin, WI, USA; d Clinical Concepts in Obstetrics, Scottsdale, AZ, USA; e Department of Statistical Science, Duke University, Durham, NC, USA; f Department of Biostatistics, University of North Carolina at Chapel Hill, Chapel Hill, NC, USA; g Department of Obstetrics, Gynecology and Reproductive Biology, Harvard Medical School, Boston, MA, USA

**Keywords:** Maternal health, Severe maternal morbidity (SMM), Risk stratification, Comorbidity index, Ordinal regression

## Abstract

**Background:**

The aim of this study is to develop and validate a maternal Prenatal Risk Index (m-PRI) comprised of preexisting maternal conditions and patient characteristics associated with increasing severity levels of severe maternal morbidity (SMM).

**Methods:**

Using administrative data from the Premier Healthcare Database, this cohort study analyzed inpatient deliveries between 2016 and 2023 across 864 hospitals and 49 U.S. states to develop the m-PRI. Multivariable ordinal logistic regression was used to quantify the association between 46 candidate conditions and an ordered set of SMM severity levels derived from an inpatient mortality-weighted SMM composite index. The final m-PRI is a composite risk score, calculated by summing the weights of existing patient conditions. The predictive performance of the m-PRI was evaluated against the Obstetric Comorbidity Scoring System (OCSS) using precision-recall area under the curve (PR-AUC) metrics overall and across subgroups.

**Findings:**

Across 7,174,412 maternal delivery hospital encounters, 56,612 patients experienced at least one SMM indicator. The final m-PRI incorporated 28 conditions. On the validation dataset, the m-PRI achieved a PR-AUC of 0.223 (95% CI: 0.194–0.254), modestly higher than the OCSS (0.173; 95% CI: 0.147–0.201), with a significant mean difference of 0.050 (95% CI: 0.036–0.064, p < 0.001). Subpopulation analysis demonstrated that PR-AUC values were consistently higher for the m-PRI than OCSS across all evaluated subgroups.

**Interpretation:**

The m-PRI improves upon existing methods by weighting maternal risk factors based on their association with mortality-informed SMM severity levels on an ordinal scale, rather than a dichotomous composite indicator. By incorporating a more expansive set of relevant maternal risk conditions, the m-PRI detects previously unmeasured risk, both overall and across clinical subgroups.

**Funding:**

The U.S. Department of Health and Human Services Office on Women’s Health supported this project under contract 75P00120C00066.

**Research in context**

**Evidence before this study**

Searches of PubMed, Embase, and Google Scholar (2000–2024) were conducted using terms related to maternal morbidity, mortality, comorbidity indices, and risk prediction, limited to the English language. Prior work showed that general indices such as Charlson and Elixhauser, as well as obstetric-specific tools, were primarily based on binary endpoints and a limited set of conditions. This reliance on dichotomous outcomes constrained their ability to capture variation in clinical severity and the full spectrum of maternal risk. These limitations highlight the opportunity to strengthen existing indices by incorporating a broader range of conditions and outcomes measured along a severity gradient.

**Added value of this study**

A maternal prenatal risk index (m-PRI) was developed that incorporated a broader set of maternal conditions and applied severity weighting. Using a nationally representative dataset, the m-PRI was shown to improve prediction of severe maternal morbidity compared with existing indices, with consistent performance across demographic and socioeconomic subgroups.

**Implications of all the available evidence**

The findings suggest that the m-PRI provides a more accurate and equitable measure of maternal risk. Incorporation into research and quality measurement could improve risk adjustment and identification of high-risk patients. Further validation in diverse settings and exploration of integration into clinical workflows are warranted.

## Introduction

While there are year-to-year fluctuations in maternal mortality, no meaningful overall improvement in the maternal mortality rate has been observed for the last half-decade.^1^, ^2^, ^3^ Compounding this maternal health challenge are rising rates of severe maternal morbidity (SMM), described by the U.S. Centers for Disease Control (CDC) as “*unexpected outcomes of labor and delivery that can result in significant short- or long-term health consequences*”, that occur at 50–60 times the rate of maternal mortality.^4^, ^5^, ^6^ In 2020, the U.S. Surgeon General’s *Call to Action* to improve maternal health emphasized the need to better understand and manage chronic conditions and advance research to better understand the interaction of comorbidities during and after pregnancy to inform appropriate interventions.^7^

There is a clear relationship between maternal morbidity, SMM rates, and maternal mortality, with the co-occurrence of morbidities being linked to higher SMM risk.^8^ This elevated risk from multiple co-morbid conditions, which arises from concurrent conditions such as infections, mental health disorders, obesity, or cardiovascular conditions, is not well understood but is recognized as a driver of adverse outcomes affecting both women and infants.^9^

Quality improvement efforts require consistent measurement of maternal risk factors, yet general comorbidity scores (Charlson, Elixhauser) have limited utility in obstetrics because they omit pregnancy-specific conditions.^10^, ^11^, ^12^, ^13^, ^14^ To address this gap, the obstetric comorbidity index (OB-CMI) was developed in 2013 using Medicaid administrative data and incorporated 20 maternal conditions, along with maternal age, to predict SMM.^15^ Additional validation studies demonstrated its potential utility for prospectively identifying SMM risk and for evaluating case-mix adjusted differences in SMM across diverse populations.^11^^,^^13^^,^^16^ Importantly, studies found that over 40% of women experiencing SMM had a maternal OB-CMI score of zero, suggesting an opportunity for further refinement through revised weighting strategies and the inclusion of additional clinically relevant conditions.^12^^,^^17^^,^^18^ Subsequent work to develop improved comorbidity scores, including the Obstetric Comorbidity Scoring System (OCSS), employed these approaches within the risk assessment framework.^11^^,^^14^^,^^19^^,^^20^

A common feature of existing maternal comorbidity indices is the use of a binary composite value of maternal mortality and/or morbidity as the endpoint for index development and validation.^14^^,^^15^^,^^19^^,^^20^ While such indices have substantially improved SMM risk adjustment, they are limited by reliance on binary SMM definitions that do not account for heterogeneity in severity or downstream harm.^21^ A recent study of over 18 million U.S. delivery hospitalizations ranked the ability of CDC SMM indicators to identify maternal mortality during the delivery hospitalization, finding that the top three indicators identified 69% of in-hospital mortality, while the bottom seven indicators combined identified just 2%.^22^ Consequently, weighting components of a comorbidity index based on their relationship to a binary SMM composite unintentionally, but inevitably, obscures the relationship between individual conditions and the severity of the clinical outcome. As a result, the ability of the resultant comorbidity index to meaningfully stratify and risk-adjust populations and accurately predict patient-level risk may be constrained.

Kilday et al. have proposed a mortality-weighted SMM index (w-SMM), expressed on a continuous scale, as a more granular metric to measure overall SMM severity compared to the current binary composite.^3^ This approach provides an opportunity to improve existing prenatal obstetric risk indices by assigning condition-specific weights that reflect their association with graduated levels of SMM severity (made available through w-SMM) rather than the current binary composite indicator. While the primary novelty of the index lies in leveraging the w-SMM outcome, the index also incorporates a wider range of maternal health factors to detect previously unmeasured risk. The aim of this study is to address the gaps outlined above by presenting a maternal Prenatal Risk Index (m-PRI) developed using a large U.S. all-payer and geographically diverse administrative dataset where the weighting of an expanded set of maternal conditions was determined using a graduated severity-adjusted (non-dichotomous) SMM measurement approach. Such an approach can improve risk stratification to support more targeted timely clinical interventions aimed at mitigating preventable SMM and improving maternal health outcomes.

## Methods

This analysis utilized data from the Premier Healthcare Database (PHD),^23^ a comprehensive, diverse all-payer administrative database comprising 25% of annual inpatient encounters in the U.S. from more than 1400 hospitals. The PHD includes detailed information on patient demographics, disease states, and services provided, such as medications, laboratory tests, and diagnostic procedures, as well as hospital characteristics. Reporting followed RECORD (an extension of STROBE) and TRIPOD guidelines.

The PHD is considered exempt from Institutional Review Board (IRB) oversight under Title 45 Code of Federal Regulations, Part 46 of the United States, specifically 45 CFR 46.101(b).^4^ In accordance with the HIPAA Privacy Rule, disclosed data from the PHD are considered de-identified per 45 CFR 164.506(d)^2^(ii) (B) through the “Expert Determination” method.

This study was conducted in accordance with the ethical principles outlined in the Declaration of Helsinki and the Belmont Report, as applicable to research using de-identified secondary data.

### Study population

This study analyzed 7,174,412 inpatient delivery encounters discharged between 2016 and 2023 among patients aged 10–64 years with a gestational age of at least 20 weeks. Delivery encounters were identified using the CDC’s SMM birth volume denominator, which uses Medicare Severity-Diagnosis Related Groups (MS-DRG) in combination with ICD-10 diagnosis and procedure codes to define inclusion and exclusion criteria. The study was limited to live and stillbirth delivery encounters and excluded encounters with an abortive outcome or unknown gestational age. ICD-10 codes used for inclusion and exclusion are provided in Supplemental Table S1. All patients had a recorded sex of female within this study according to the Uniform Billing (UB-04) standard.

### Study variables

All clinical conditions were identified using ICD-10 diagnosis and procedure codes from the patient discharge record. A targeted literature review was conducted to identify candidate conditions using PubMed, prioritizing studies of validated prediction models assessing maternal mortality and morbidity outcomes. The conditions included in the finalized 2022 OCSS composite version, which was calibrated against the most current CDC SMM definition that excludes blood transfusion, served as the foundational reference for candidate conditions. Additional conditions were abstracted and consolidated across sources, informed in part by the Clinical Classifications Software Refined (CCSR) framework^24^ and screened for clinical relevance and feasibility of measurement in administrative data. Clinicians and clinical coding experts reviewed all condition definitions and refined inclusion and exclusion criteria to ensure accurate capture of relevant conditions.

Of the 27 candidate conditions in the OCSS composite (25 in the final composite), 10 were modified, resulting in 13 updated conditions. Such modifications were introduced to provide a more granular definition of an existing condition or expand the list of ICD-10 codes used to define it. Of special note, given the heterogeneity of BMI ICD-10 coding in administrative data, a broader definition of obesity was defined. While this modification improves case capture, it may also underestimate risk among the most severely obese patients. The full rationale behind these modifications is further described in Supplemental Table S2. Additionally, the m-PRI evaluated an expanded set of conditions and characteristics for inclusion in the index, including assisted reproductive technology, bariatric surgery, cancer, hepatitis infection, immune disorders, infection of the amniotic sac and membranes, infections commonly associated with sexual transmission, neurologic disorders, non-sexually transmitted infections, oligohydramnios, other endocrine conditions (e.g., disorders of thyroid, lipid metabolism, and pituitary gland), other renal conditions (e.g., dialysis, nephritis, nephrosis, and renal sclerosis), preterm premature rupture of membranes (PPROM), other ruptured membranes, polyhydramnios, and maternal age of 19 years or younger. To distinguish preexisting conditions from SMM, bleeding disorders, non-sickle cell anemia, and sickle cell anemia/trait required their associated ICD-10 codes to be present on admission (POA) or POA-exempt. ICD-10 definitions for each condition are provided in Supplemental Table S3. Given this study used administrative data, all variables were coded with no explicitly missing values.

To develop the m-PRI, the primary outcome of interest was a mortality-weighted SMM composite based on the 20 CDC-defined SMM indicators.^3^^,^^25^ This continuous weighted measure incorporated each indicator’s conditional probability of inpatient mortality and was used to derive composite weights, with higher weights indicating greater risk of inpatient mortality during the delivery encounter.

### Model development

Multivariable ordinal logistic regression was employed to quantify the relationship between the evaluated maternal conditions and the mortality-weighted SMM index. The weighted SMM index followed a highly zero-inflated distribution, with approximately 99% of the values equal to zero, given the rarity of SMM. To enhance interpretability and enable ordinal modeling, the composite was binned into four distinct severity groups, referred to as SI levels 1 through 4. SI level 1 represented no SMM (SMM index = 0). Cut points for the remaining SI levels, 2 through 4, were computed using k-means clustering and further refined by visual inspection to improve the separation of the multimodal distribution of the SMM index. Using the final cut points, SI level 1 included values with an SMM index of zero, SI level 2 included values greater than 0 and less than or equal to 0.018, SI level 3 included values between 0.018 and 0.064, and SI level 4 included values greater than 0.064. Supplemental Fig. S1 displays the distribution of the SMM index values greater than 0 and less than 0.2, accounting for 98% of the SI level 2 to 4 values, along with the corresponding SI level breakpoints.

Prior to modeling, collinearity among conditions was assessed through pairwise correlations. Any two variables exceeding an arbitrarily defined Pearson correlation coefficient threshold of 0.4 were resolved by exclusively coding the condition downstream on the causal pathway when both conditions were present. Additionally, variance inflation factors for each condition were assessed to limit multicollinearity. The proportional odds assumption was assessed using Brant–Wald testing and inspection of cumulative log-odds across severity levels.

Although no variance inflation factors exceeded 2, the correlation matrices revealed two sets of variables with moderate correlations (greater than 0.4). For these pairs, the variable occurring downstream in the clinical pathway was retained in instances where both variables were present. For the first pair, preeclampsia with severe features and chronic hypertension, the m-PRI model was adjusted to set chronic hypertension to 0 if both variables were present. Similarly, for PPROM and pre-term delivery, PPROM was set to 0 if both variables were present.

The m-PRI was constructed as a composite of these condition indicators, with weights assigned based on their respective regression coefficients. Although a single ordinal regression model was fit, each candidate condition represented a distinct hypothesis regarding its association with increasing SMM severity. As such, p-values were adjusted using the Bonferroni-Holm method to account for multiple comparisons.

Consistent with prior methods, conditions with adjusted p-values exceeding 0.05 or those with negative coefficients were excluded before formulating the m-PRI to retain only those demonstrating positive statistical significance at the 5% alpha level. Given that the candidate variables were selected based on their biological plausibility and are established risk factors for maternal outcomes, negative coefficients were dropped due to potential unresolved collinearity, given the candidate conditions likely do not have a protective effect with SMM. Additionally, the use of adjusted p-values helped ensure that only conditions with statistically robust associations were retained, minimizing the influence of small estimates that could artificially inflate the relative impact of the other conditions based on the scaling of the m-PRI.

To construct the m-PRI, weights for each retained condition j were scaled by dividing the regression coefficient $\beta_{j}$ by the minimum estimate $\beta_{\min}$ to obtain a point value for greater interpretation, consistent with the approach used in the OCSS.^20^^,^^26^^,^^27^ The point value was rounded to obtain the weighted score $w_{j}$ for each condition. This approach allows weights to be interpreted as a multiplicative difference in risk relative to the smallest significant risk factor. The smallest risk factor will be assigned a score of 1, given $\beta_{\min}$ will equal its regression coefficient.

$$w_{j}=round\left(\frac{\beta_{j}}{\beta_{\min}}\right)$$

For each patient i, the m-PRI was calculated as the sum of scaled weights $w_{j}$ corresponding to each maternal condition present $x_{j,}$. Regression coefficients and point values for each condition are provided in Table 1.

$${mPRI}_{i}=\sum_{j=1}^{p}w_{j}x_{j,i}$$

**Table 1:** Maternal risk factors and model weights.

Maternal risk factor	Included in m-PRI	Included in OCSS	Prevalence		Log odds	95% Lower CI	95% Upper CI	Adj. P value	m-PRI weight
			Count	%					
Placenta accreta spectrum	Yes	Yes^a^	9188	0.1	4.19	4.14	4.24	<0.0001	28
Chronic renal disease	Yes	Yes	19,422	0.3	2.55	2.51	2.60	<0.0001	17
Acquired cardiac disease	Yes	Yes^a^	73,082	1.0	1.97	1.93	2.00	<0.0001	13
Pulmonary hypertension	Yes	Yes	1924	<0.1	1.61	1.50	1.72	<0.0001	11
Infection of amniotic sac and membranes	Yes	No	155,191	2.2	1.54	1.51	1.58	<0.0001	10
Preeclampsia with severe features	Yes	Yes	298,798	4.2	1.20	1.17	1.22	<0.0001	8
Neurologic disorders	Yes	No	61,996	0.9	1.18	1.14	1.22	<0.0001	8
Asthma (acute or moderate/severe)	Yes	Yes	432,794	6.0	1.16	1.14	1.18	<0.0001	8
Neuromuscular disease	Yes	Yes	36,520	0.5	1.05	0.99	1.11	<0.0001	7
Pre-term birth (less than 37 weeks)	Yes	Yes^a^	757,329	10.6	1.00	0.98	1.02	<0.0001	7
Placenta previa (complete or partial)	Yes	Yes^a^	34,638	0.5	0.81	0.75	0.86	<0.0001	5
Bleeding disorder (preexisting)	Yes	Yes	156,071	2.2	0.78	0.75	0.82	<0.0001	5
Other endocrine conditions	Yes	No	418,746	5.8	0.72	0.70	0.75	<0.0001	5
Placental abruption	Yes	Yes	78,842	1.1	0.72	0.67	0.76	<0.0001	5
Cancer	Yes	No	25,161	0.4	0.69	0.61	0.78	<0.0001	5
Hepatitis infection	Yes	No	47,895	0.7	0.53	0.46	0.60	<0.0001	3
Gastrointestinal disease	Yes	Yes	115,821	1.6	0.49	0.44	0.53	<0.0001	3
Non-sexually transmitted infections	Yes	No	1,518,829	21.2	0.47	0.45	0.49	<0.0001	3
Pre-eclampsia without severe features or gestational hypertension	Yes	Yes	705,717	9.8	0.37	0.34	0.39	<0.0001	2
Non-sickle cell anemia	Yes	Yes^a^	942,096	13.1	0.37	0.35	0.39	<0.0001	2
Assisted reproductive technology	Yes	No	7612	0.1	0.32	0.15	0.49	0.003	2
Uterine fibroids	Yes	Yes	124,498	1.7	0.30	0.25	0.35	<0.0001	2
Chronic hypertension	Yes	Yes	59,192	0.8	0.28	0.21	0.36	<0.0001	2
Previous cesarean birth	Yes	Yes^b^	1,284,879	17.9	0.24	0.22	0.26	<0.0001	2
Multiple gestation	Yes	Yes	127,827	1.8	0.23	0.18	0.27	<0.0001	2
Substance use disorder	Yes	Yes^a^	477,134	6.7	0.22	0.19	0.25	<0.0001	1
Age 35 and older	Yes	Yes	1,316,829	18.4	0.20	0.18	0.22	<0.0001	1
Age 19 and younger	Yes	No	334,420	4.7	0.15	0.11	0.19	<0.0001	1
COVID-19 infection	Control	No							9^c^
Immune disorders	No	No	2922	<0.1	0.20	0.00	0.40	0.63	
Type I preexisting diabetes	No	Yes^a^	23,094	0.3	0.10	0.02	0.18	0.26	
Congenital cardiac disease	No	Yes^a^	7304	0.1	0.10	−0.03	0.22	1	
Preterm premature rupture of membranes (PPROM)	No	No	9502	0.1	0.09	−0.18	0.36	1	
HIV/AIDS	No	Yes	5858	0.1	0.05	−0.15	0.26	1	
Thyrotoxicosis	No	Yes	20,448	0.3	0.04	−0.08	0.16	1	
Sickle cell anemia or trait	No	Yes^a^	51,719	0.7	0.03	−0.04	0.11	1	
Sexually transmitted infections	No	No	333,022	4.6	0.03	0.00	0.06	0.99	
Other ruptured membranes	No	No	458,840	6.4	−0.01	−0.05	0.04	1	
Obesity	No	Yes^b^	994,298	13.9	−0.01	−0.04	0.01	1	
Connective tissue or autoimmune disease	No	Yes	16,488	0.2	−0.02	−0.13	0.08	1	
Polyhydramnios	No	No	129,503	1.8	−0.03	−0.09	0.02	1	
Major mental health disorder	No	Yes^a^	644,974	9.0	−0.05	−0.08	−0.02	0.002	
Gestational diabetes mellitus	No	Yes	630,612	8.8	−0.07	−0.09	−0.04	0.0002	
Other renal disease	No	No	503	0.0	−0.11	−0.35	0.13	1	
Oligohydramnios	No	No	179,915	2.5	−0.13	−0.18	−0.07	<0.0001	
Type II or unspecified preexisting diabetes	No	Yes^a^	76,658	1.1	−0.17	−0.22	−0.11	<0.0001	
Bariatric surgery	No	No	36,445	0.5	−0.37	−0.47	−0.27	<0.0001	

a Modified OCSS measure.

b Included in original OCSS score when including blood transfusions for SMM, but omitted when excluding them.

c For retrospective usage during peak COVID-19 time periods.

Given its potential confounding effects, COVID-19 was included in the model used to derive condition weights; however, with its diminishing severity in recent years, no weight was assigned to COVID-19 for the prospective use of the index. Excluding the COVID-19 indicator mitigates the potential inflation of COVID-19 risk, given that the weight was derived during a period in which COVID-19 was associated with greater severity. The weight associated with COVID-19 has been provided in Table 1 to support retrospective analyses.

### Evaluating model performance

Predictive performance between the m-PRI and OCSS was compared against binary SMM and ordinal SMM outcomes. First, given that the OCSS was designed to estimate the risk of SMM based on the occurrence of any single indicator, the ability of the m-PRI and OCSS to predict the binary occurrence of SMM was assessed. In this scenario, a set of multivariable logistic regression models was fit to assess the variation explained in the binary occurrence of SMM by the respective m-PRI and OCSS indices. Out-of-sample predictive performance was evaluated through temporal validation, where data from 2016 to 2022 served as the derivation dataset and 2023 was withheld as the validation dataset. This approach accounts for the varying non-stationary nature of the data, driven by the fluctuating prevalence and severity of COVID-19 throughout the study period. Covariate distributions were evaluated using standardized mean differences (SMD) to ensure comparability between the derivation and validation datasets. Only Hispanic ethnicity showed meaningful differences when applying a 0.2 SMD threshold.

Using the validation dataset, area under the receiver operating characteristic curve (ROC-AUCs) values were calculated for each logistic regression model, and ROC curves were generated. The performance of both models was further evaluated using a DeLong test to compare ROC-AUCs, using a significance level of 5%. Precision-recall curves (PR-AUCs) were also calculated using the validation dataset as a balancing measure for both models. Bootstrapping was used to assess differences between the PR-AUCs in model performance. A total of 100,000 deliveries were resampled with replacement from the validation set 5000 times, and the resulting distribution of PR-AUC differences was used to determine statistical significance.^28^ Additionally, 95% bootstrap confidence intervals were generated for each PR-AUC estimate. For the m-PRI, model calibration was assessed using a calibration plot comparing observed versus predicted SMM probabilities, complementing the evaluation of discrimination metrics. Subgroup PR-AUC values were calculated across maternal age, race, and payer to evaluate model bias and generalization.

Second, ordinal models were fit using SMM severity level as the outcome, with the m-PRI and OCSS composite scores evaluated separately as predictors. To account for the highly imbalanced distribution across severity levels and the clinical preference for over-identification, inverse frequency weights were applied to each ordinal group.^29^ Although the OCSS was intended for binary SMM assessment, this analysis is intended to demonstrate the application of an ordinal SMM outcome, rather than to establish comparative superiority between indices.

Predicted score distributions were summarized using multiclass confusion matrices (Supplemental Figs. S2 and S3) and severity level precision, recall, and F1 statistics (Supplemental Table S4). To further characterize the relationship between the m-PRI scores and severity levels, a box plot comparing the m-PRI distributions across severity groups is provided in Supplemental Fig. S4.

### Sensitivity analysis

To further disentangle performance differences attributable to changes in the outcome definition, proposed OCSS coding modifications, and the inclusion of additional risk factors, PR-AUCs were assessed under two alternative scenarios. In the first scenario, the original OCSS ICD-10 definitions were retained (including candidate conditions), and the composite was reweighted based on the ordinal weighted SMM outcome. In the next scenario, only the set of modified and unmodified OCSS risk factors was evaluated. These two models were compared with both the baseline (i.e., the published OCSS weights) and final m-PRI composite.

All analyses were conducted using R statistical software, Version 4.4.1 (R Foundation for Statistical Computing).^30^ Ordinal logistic regression models were fit using the MASS library.^31^

### Role of the funding source

The study was funded by the U.S. Department of Health and Human Services (HHS) Office on Women’s Health (OWH) under contract 75P00120C00066. Representatives of the funding organization are included among the co-authors and contributed to study design and writing of the report. The remaining authors independently conducted data collection, data analysis, and data interpretation as well as contributing to study design, drafting, and revising of the manuscript.

## Results

### Patient characteristics

The final study population included 7,174,412 maternal delivery visits from 864 U.S. hospitals across 49 states and the District of Columbia. The initial cohort included 66,722 additional pregnancies that were dropped due to missing gestational age. Twenty-seven percent (n = 1,913,216) of births occurred in a council of teaching hospital (COTH) facility, with 17% (n = 1,198,219) of deliveries in hospitals with less than 1000 annual deliveries, 23% (n = 1,651,585) in hospitals with 1000–1999 deliveries, 41% (n = 2,914,962) in hospitals with 2000–3999 deliveries, and 20% (n = 1,409,646) in hospitals with 4000 or more deliveries. The study population consisted of 63% (n = 4,540,146) White, 15% (n = 1,074,767) Black, 5% (n = 334,958) Asian, and 1% American Indian/Alaska Native (n = 61,080) individuals, with a mean age of 29.1 (SD = 5.8). The women in the analysis primarily had either commercial (52%; n = 3,726,993) or Medicaid (42%; n = 3,027,548) insurance. Of the overall study population, 6,341,166 were included in the derivation dataset and 833,246 in the validation dataset. The validation dataset included a higher proportion of reported Hispanic or Latino ethnicity (22%; n = 183,796) compared to the overall cohort (18%; n = 1,280,737). Patient characteristics for the derivation and validation datasets are provided in Supplemental Table S5.

The overall SMM rate within the study population was 1% (n = 56,612). Of the 56,612 patients with SMM, 74% (n = 41,632) were categorized as level 2, 18% (n = 10,310) as level 3, and 8% (n = 4670) as level 4. The mean SMM Index was 0.01 (SD < 0.01) for level 2, 0.03 (SD = 0.01) for level 3, and 0.16 (SD = 0.14) for level 4. Table 2 contains the patient characteristics for the study population, overall and by SI level. Differences were identified in prevalence among racial groups across the SI levels. Black women were disproportionately represented in higher SMM severity groups, representing 23% (n = 9519) in level 2, 22% (n = 2244) in level 3, and 24% (n = 1101) in level 4 compared to 15% (n = 1,074,767) within SI level 1. Similar patterns were observed in the Medicaid population, where 53% (n = 2474) of women in the level 4 SI group were insured by Medicaid compared with 42% (n = 3,027,548) in SI level 1. Differences were also observed in delivery mode across SI levels. In SI level 1, 32% (n = 2,291,428) of women had a Cesarean delivery, while more than 65% (n = 38,472) of women in levels 2, 3, and 4 delivered via Cesarean section.

**Table 2:** Patient and hospital characteristics by SMM index severity group.

Characteristics	Overall		SI Level I		SI Level 2		SI Level 3		SI Level 4		p value	SMD
	N	%	n	%	N	%	n	%	n	%		
Total N	7,174,412		7,117,800		41,632		10,310		4670			
Maternal age											<0.001	0.165
Age (mean/SD)	29.06/5.78		29.05/5.78		30.22/6.34		30.56/6.31		30.97/6.45			
Race											<0.001	0.154
American Indian/Alaska Native	61,080	0.90%	60,420	0.80%	466	1.10%	129	1.30%	65	1.40%		
Asian	334,958	4.70%	332,128	4.70%	1972	4.70%	643	6.20%	215	4.60%		
Black	1,074,767	15.00%	1,061,903	14.90%	9519	22.90%	2244	21.80%	1101	23.60%		
Other	746,533	10.40%	740,343	10.40%	4533	10.90%	1136	11.00%	521	11.20%		
Pacific Islander	71,904	1.00%	71,232	1.00%	446	1.10%	169	1.60%	57	1.20%		
Unknown	345,024	4.80%	342,131	4.80%	2124	5.10%	522	5.10%	247	5.30%		
White	4,540,146	63.30%	4,509,643	63.40%	22,572	54.20%	5467	53.00%	2464	52.80%		
Ethnicity											0.009	0.034
Hispanic or latino	1,280,737	17.90%	1,270,534	17.90%	7501	18.00%	1900	18.40%	802	17.20%		
Not hispanic or latino	4,856,503	67.70%	4,818,505	67.70%	28,003	67.30%	6899	66.90%	3096	66.30%		
Unknown	1,037,172	14.50%	1,028,761	14.50%	6128	14.70%	1511	14.70%	772	16.50%		
Payer											<0.001	0.151
Charity or indigent	8727	0.10%	8652	0.10%	56	0.10%	11	0.10%	8	0.20%		
Commercial insurance	3,726,993	51.90%	3,701,129	52.00%	19,485	46.80%	4496	43.60%	1883	40.30%		
Medicaid	3,027,548	42.20%	3,000,431	42.20%	19,433	46.70%	5210	50.50%	2474	53.00%		
Medicare	40,336	0.60%	39,399	0.60%	679	1.60%	172	1.70%	86	1.80%		
Other/unknown	370,808	5.20%	368,189	5.20%	1979	4.80%	421	4.10%	219	4.70%		
Admission type											<0.001	0.205
Elective	3,853,433	53.70%	3,827,874	53.80%	19,671	47.20%	4180	40.50%	1708	36.60%		
Unknown	292,694	4.10%	290,809	4.10%	1363	3.30%	358	3.50%	164	3.50%		
Trauma/emergency/urgent	3,028,285	42.20%	2,999,117	42.10%	20,598	49.50%	5772	56.00%	2798	59.90%		
Admission source											<0.001	0.241
Unknown/other	67,266	0.90%	66,702	0.90%	407	1.00%	111	1.10%	46	1.00%		
Clinic	1,310,680	18.30%	1,300,884	18.30%	7500	18.00%	1638	15.90%	658	14.10%		
Non-healthcare facility	5,700,086	79.50%	5,656,870	79.50%	31,908	76.60%	7874	76.40%	3434	73.50%		
Transfer from other healthcare facility	96,380	1.30%	93,344	1.30%	1817	4.40%	687	6.70%	532	11.40%		
Discharge status											<0.001	0.451
Expired	433	<0.1%	17	<0.1%	7	<0.1%	18	0.20%	391	8.40%		
Home/home health	7,147,474	99.60%	7,093,572	99.70%	40,627	97.60%	9725	94.30%	3550	76.00%		
Other/unknown	11,135	0.20%	10,495	0.10%	364	0.90%	182	1.80%	94	2.00%		
Transfer	15,370	0.20%	13,716	0.20%	634	1.50%	385	3.70%	635	13.60%		
Delivery type											<0.001	0.487
Cesarean	2,329,900	32.50%	2,291,428	32.30%	27,517	66.10%	7466	72.40%	3489	74.70%		
Vaginal	4,844,512	67.50%	4,826,372	67.80%	14,115	33.90%	2844	27.60%	1181	25.30%		
Severe maternal morbidity												
SMM index (mean/SD)	<0.01/0.01		<0.01/<0.01		0.01/<0.01		0.03/0.01		0.16/0.14			
Maternal mortality												

### Model inputs

The most prevalent maternal candidate conditions in the study population were non-sexually transmitted infection (21%; n = 1,518,829), previous Cesarean birth (18%; n = 1,284,879), obesity (14%; n = 994,298), and non-sickle cell anemia (13%; n = 942,096). Additionally, 18% (n = 1,316,829) of the population was identified as 35 years or older, while 5% (n = 334,420) was 19 or younger, thus capturing risk for women of relatively older and younger reproductive age.

Of the 46 candidate risk factors evaluated, 28 met the inclusion criteria in the m-PRI model (Table 1). Among the included risk factors, 13 intersected with those used in the OCSS, 7 were variants of OCSS conditions using modified definitions, and 8 were new conditions. Placenta accreta spectrum (β = 4.19, 95% CI: 4.14–4.24), chronic renal disease (β = 2.55, 95% CI: 2.51–2.60), and acquired cardiac disease (β = 1.97, 95% CI: 1.93–2.00) were identified as conferring the highest risk of SMM, with m-PRI component scores of 28, 17, and 13, respectively. Eight new conditions were significantly associated with SMM severity and included in the m-PRI. Of these, infection of the amniotic sac and membranes (β = 1.54, 95% CI: 1.51–1.58), neurologic disorders (β = 1.18, 95% CI: 1.14–1.22), and other endocrine disorders (β = 0.72, 95% CI: 0.70–0.75) had the highest impact on SMM risk. Weights for each risk factor are provided in Table 1. In the validation sample, m-PRI scores ranged between 0 and 84.

### Model performance

**Fig. 1:**
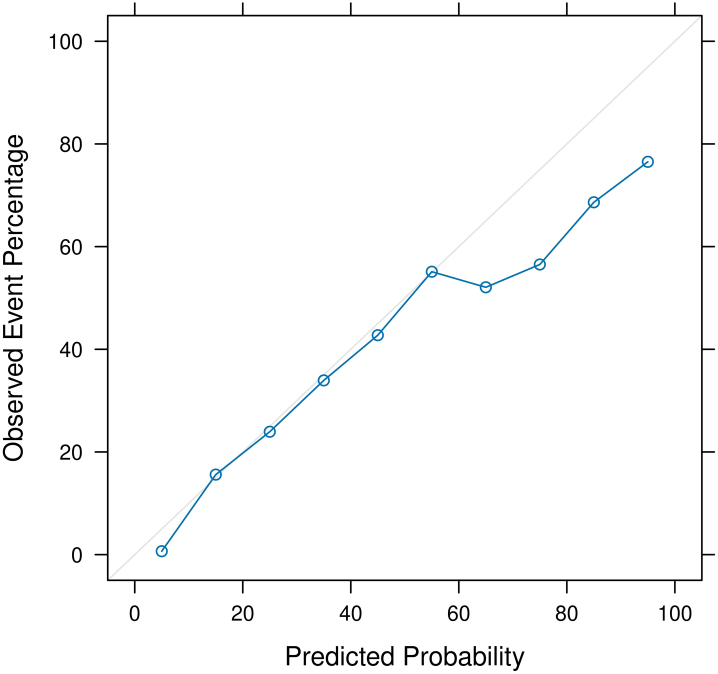
m-PRI calibration curve.

Mean m-PRI scores for the validation sample increased monotonically over SI levels with a mean of 5.07 (SD = 5.88) in level 1, 19.76 (SD = 14.75) in SI level 2, 23.39 (SD = 15.40) in SI level 3, and 29.70 (SD = 18.23) in SI level 4 (p < 0.0001) (Supplemental Fig. S4). A calibration plot illustrating actual versus predicted SMM levels demonstrated that while m-PRI exhibits an acceptable overall fit, the model overestimated SMM risk for high-risk patients (Fig. 1).

**Fig. 2:**
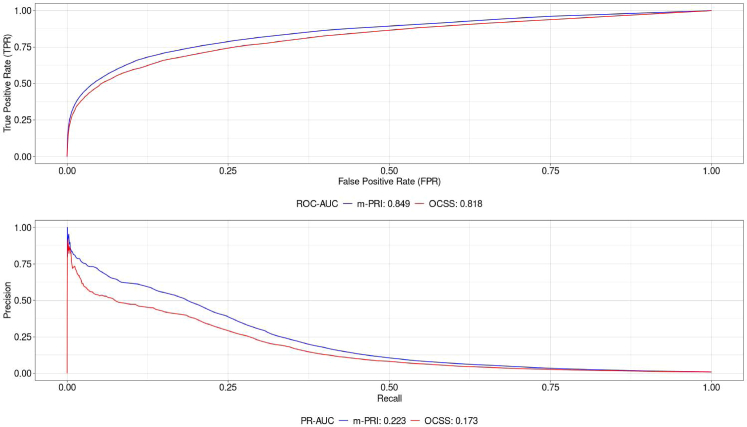
ROC and PR-AUC for m-PRI and OCSS.

Of 7897 women with SMM in the validation sample, 97% (n = 7626) had a nonzero m-PRI score indicating elevated risk of SMM, while 91% (n = 7213) had a nonzero score using the OCSS. As shown in Fig. 2, the ROC-AUC for the m-PRI was modestly higher than OCSS (0.849 versus 0.818, p < 0.0001) when predicting SMM, with a DeLong-estimated mean difference of 0.031 (95% CI: 0.027–0.034, p < 0.0001), indicating an incremental improvement in overall ability to correctly classify positive and negative cases across all thresholds. Since SMM is a rare event, discriminatory power for the m-PRI was further evaluated using the PR-AUC. The m-PRI achieved a PR-AUC of 0.223 (95% CI: 0.194–0.254) compared with 0.173 (95% CI: 0.147–0.201) for OCSS. The mean PR-AUC difference was 0.050 (95% CI: 0.036–0.064, p < 0.001), demonstrating greater discriminatory performance in identifying high-risk cases while balancing precision and recall.

As part of the sensitivity analyses, PR-AUCs were evaluated for two alternative model specifications. Reweighting the original OCSS candidate conditions using the weighted SMM outcome increased the PR-AUC from 0.173 to 0.194, corresponding to an absolute increase of 0.021 (Supplemental Table S6). When the model was restricted to the set of modified and unmodified OCSS candidate conditions and similarly reweighted, the PR-AUC increased further to 0.206, resulting in an additional increase of 0.012.

PR-AUC scores were calculated for the m-PRI and OCSS within sub-populations across maternal age, race, and payer to evaluate model bias and generalization. The m-PRI demonstrated greater discriminatory power in predicting SMM compared to OCSS across the majority of the subpopulations. Model performance for both the m-PRI and OCSS varied within sub-populations, with higher PR-AUC scores among sub-populations experiencing elevated rates of SMM (Table 3).

**Table 3:** PR-AUC by patient demographic characteristics.

Characteristics	SMM Rate	OCSS	m-PRI
Maternal age			
10–19	0.8%	0.113	0.185
20–24	0.6%	0.126	0.162
25–29	0.7%	0.139	0.181
30–34	0.8%	0.182	0.231
35–39	1.1%	0.204	0.267
40–44	1.6%	0.260	0.315
45–64	2.8%	0.334	0.370
Maternal race			
American Indian/Alaska Native	1.3%	0.279	0.343
Asian	1.1%	0.128	0.192
Black	1.4%	0.223	0.269
Other	1.0%	0.194	0.257
Pacific Islander	1.0%	0.190	0.263
Unknown	1.1%	0.136	0.202
White	0.8%	0.156	0.202
Payer type			
Charity or indigent	0.9%	0.397	0.279
Commercial insurance	0.8%	0.147	0.190
Medicaid	1.1%	0.200	0.262
Medicare	3.2%	0.324	0.331
Other/Unknown	0.8%	0.127	0.162

Predicted score distributions across the four SMM severity levels were examined for both the m-PRI and OCSS composites (Supplemental Table S4). For SI level 1, which represents the absence of an SMM event, both indices exhibited high precision (m-PRI: 0.997, OCSS: 0.996) and recall (m-PRI: 0.792, OCSS: 0.806). For SI levels 2 and 3, precision ranged from 0.008 to 0.013, and recall ranged from 0.167 to 0.307. The rarest and most severe level (SI level 4) had higher precision (m-PRI: 0.031, OCSS: 0.017) and recall (m-PRI: 0.490, OCSS: 0.508) compared with SI levels 2 and 3. F1 scores reflected these distributions.

## Discussion

The m-PRI was developed using a large, geographically diverse all-payer administrative dataset. Candidate maternal conditions were drawn from prior literature and existing comorbidity indices, then refined for clinical relevance. Multivariable ordinal logistic regression was used to evaluate these conditions, and condition-specific weights were derived based on a mortality-weighted SMM outcome that captured a spectrum of severity rather than a simple binary event. Each patient’s m-PRI score is the sum of weighted conditions present, providing a clear and interpretable measure of prenatal maternal risk. Of the 46 evaluated maternal clinical conditions, 28 satisfied the variable selection criteria to be included as components in the final m-PRI. Of the 28 retained conditions, 13 directly aligned with those defined in the OCSS, 7 overlapped with OCSS conditions but with modified definitions, and 8 were new conditions unique to the m-PRI.

The m-PRI retained most of the heavily weighted conditions in the OCSS, while incorporating additional clinically relevant risk factors, such as infection of amniotic sac and membranes and younger maternal age. Several OCSS conditions did not satisfy the variable selection criteria, including HIV/AIDS, gestational diabetes, and connective tissue or autoimmune disease. The prevalence of these conditions in the study population was similar to that reported in the OCSS development dataset, except for HIV/AIDS. These findings do not diminish the clinical importance of these conditions but rather reflect potential differences in their distribution across SMM events of varying severity. This illustrates that the m-PRI is intended as a composite risk score, with individual condition weights reflecting their predictive contribution to severity-adjusted SMM risk, rather than independent causal effects.

The m-PRI demonstrated reasonable ability to predict SMM, both when defined using graduated severity levels and as a dichotomous indicator, exhibiting a clear dynamic range and differentiation across the four SMM Index risk levels.

Direct performance comparison with the OCSS to predict the binary occurrence of SMM revealed that the m-PRI had a statistically significant higher ROC-AUC and PR-AUC than the OCSS for the overall validation population. In addition, to validate the general applicability of the m-PRI for risk-adjusting differing populations, whether within or across facilities or over time, performance between the m-PRI and the OCSS for sub-populations delineated by race, binned maternal age, and payer type was compared. In each sub-population evaluated, the m-PRI demonstrated a higher PR-AUC relative to the OCSS score.

The improved performance of the m-PRI against the OCSS is likely due to various reasons highlighted by the sensitivity analysis. Reweighting the composites based on a severity-weighted SMM outcome allowed the index to assign more precise risk contributions across the spectrum of SMM, in comparison to a purely binary composite SMM definition. Second, modifications to certain OCSS conditions arguably improved their representation of clinically relevant risk factors as seen by the incremental increase in the PR-AUC estimate. Finally, the inclusion of an expanded set of risk factors compared to the OCSS further expanded the index’s scope, capturing additional dimensions of maternal risk. Together, these features may help explain the observed improvements in predictive performance.

In examining multiclass outcomes, shown through paired confusion matrices, overall performance across both models remained limited. Predicting SMM events and their severity levels presents considerable challenges due to their low prevalence, resulting in highly imbalanced data in which most patients are non-SMM cases and only a small fraction have an SMM event. This imbalance can lead to poor sensitivity or overfitting to the majority class. Furthermore, the clinical presentation of SMM is often heterogeneous, and risk may be shaped by complex, interacting factors not fully captured in the data. Nevertheless, this analysis demonstrated the potential value of modeling SMM beyond its binary measure, particularly as an ordinal outcome, providing additional insight into risk gradients and severity patterns. Even modest improvements in predictive performance, particularly in recall and F1 score for rare outcomes, represent meaningful progress for the early identification of high-risk patients to inform targeted clinical interventions.

### Clinical implications

The utility of the m-PRI lies in providing an improved and generalizable method for identifying increasing SMM risk within maternal populations based on a composite score. The m-PRI may serve as an input to risk-adjust outcomes, a method to stratify high-risk patient populations, or a prospective mechanism for point of care interventions.^20^ For example, prior analyses have suggested the OB-CMI could support patient-level risk stratification, for anesthesia escalation in Cesarean deliveries, SMM risk, or as an early warning signal for patients who may warrant increased surveillance and targeted preventive care.^16^^,^^32^^,^^33^

However, translation of the OB-CMI into routine clinical decision-making has been limited and produced mixed results. When used to risk-stratify and triage transfers to a level IV maternal care facility, it accurately identified many low-risk patients (high specificity) but did not capture a meaningful proportion of patients who may have required higher-level care.^34^ In a high-acuity labor and delivery setting, incorporation of OB-CMI into standard workflows to promote risk awareness and targeted responses did not translate into lower rates of SMM.^35^ As the m-PRI showed improved performance over the OCSS (which had better performance than OB-CMI) for identifying high-risk patients in retrospective testing, the m-PRI may provide greater utility for patient-level care in clinical practice.

### Strengths

This work has several strengths. The dataset employed in this study reflects over 7.1 million maternal visits occurring in hospitals across 49 U.S. states and the District of Columbia, including over 56,000 cases of non-transfusion SMM. The nationally representative all-payer database has a robust range of patients from hospitals and healthcare systems with diverse structural and operational characteristics. The m-PRI incorporates an expanded set of clinically relevant risk factors and uses a severity-weighted SMM index to assign risk, providing an interpretable and novel prediction framework. The index demonstrates applicability across multiple subpopulations, supporting its potentially utility for broad clinical risk stratification and early identification of high-risk patients.

### Limitations and future work

ICD-10 coding specificity and consistency may vary by hospital and, therefore, is subject to potential information bias. Additionally, both over- and under-coding are plausible with data derived from administrative records, particularly for conditions such as chorioamnionitis, for which histopathologic confirmation is not routinely available. While the study controlled for COVID-19 as a confounder, with deliberate testing of performance against a post-pandemic population from 2023, the secondary effects of the COVID-19 pandemic may have influenced the care of patients during that time, potentially distorting the coded etiology of patients with different clinical conditions progressing to SMM relative to the pre- or post-pandemic care environment. Although COVID-19 was excluded from the final m-PRI score, its inclusion, as well as the other conditions that didn’t meet final inclusion criteria, as a covariate in the fitted model may indirectly influence the estimated weights of the other m-PRI conditions. While the impact is expected to be limited, this should be considered when interpreting condition-specific weights post-pandemic.

While the m-PRI provides an improved composite for discriminating SMM, its primary focus is on predictive performance using an expanded set of conditions and a weighted SMM measure. This composite does not imply causal relationships between individual conditions and SMM, even though steps were taken to reduce multicollinearity. Rather, it provides an interpretable score that can be applied at the point of care or retrospectively to predict not only SMM risk but also graduated mortality risk based on the weighted SMM. For example, many comorbidities are associated with one of the composite risk factors, preterm birth, which may obscure the true total effect of each individual risk factor on SMM and limit the interpretability of their individual contributions. Other pregnancy-related factors, such as gestational age, have been shown in prior to studies to increase the risk of SMM. Exploring these relationships by examining potential mediation or effect modification between preexisting comorbidities and SMM could provide additional insights into maternal risk. Furthermore, the m-PRI is calibrated against severity-adjusted SMM occurring during the delivery hospitalization. Further work could explore alternative weighting strategies, such as the disutility-based harm weighting used in the AHRQ PSI-90 composite.^36^ Given the relevance of SMM and mortality to the broader postpartum episode, additional research could explore recalibrating condition weights against broader cross-continuum episodic outcomes.

Additionally, while the m-PRI calibration curve showed strong overall calibration, it modestly overestimates risk among high-risk patients. This conservative overestimation may reduce the risk of missed opportunities for intervention at the expense of unnecessary intervention.

While the m-PRI was developed using U.S. administrative data and coding standards, direct transferability to other countries may be limited by differences in coding practices, health systems, and definitions of SMM. However, the underlying methodological framework, which derives a composite index using a graduated rather than dichotomous maternal morbidity measure, may be applicable to other settings despite region-specific coding standards. For example, many countries use a dichotomous measure of severe acute maternal morbidity (SAMM), and prior work has demonstrated substantial variation in severity across SAMM indicators.^37^ Adopting a weighted non-dichotomous framework could enhance discrimination and risk stratification in non-U.S. contexts.

Finally, because the highest-acuity patients are more likely to require inter-facility transfer, inclusion of transferred patients may bias risk estimates among the highest-risk group. However, excluding transferred patients or modeling transfer as a covariate would introduce greater bias by preferentially removing or reclassifying the sickest patients and conflating underlying maternal risk with health system processes, thereby reducing the interpretability and prospective usefulness of the index.

### Conclusion

An innovative maternal prenatal risk index designed to identify patients vulnerable to SMM was developed and validated in this study. Building on the foundational work by Leonard et al. and other prior efforts to develop maternal comorbidity indices, this work extends existing approaches by 1) deriving risk factor weights using a graduated, rather than binary, SMM severity framework and 2) evaluating and incorporating a more expansive set of maternal conditions and characteristics as risk factors for SMM. The m-PRI exhibited consistently improved predictive performance relative to existing risk scores, both on the overall study population and across sub-populations. As such, it has potential utility for risk-adjusting maternal health outcomes and as an index to stratify high-risk patient populations. In addition to its usage in retrospective analyses, the parsimonious nature of the m-PRI and its straightforward additive computation (i.e., assigning and summing weights for each condition present) supports its practical feasibility as a clinical decision support tool for prospective risk assessment of obstetric patients.

## Contributors

Conceptualization, D.K., D.T., M.K., K.B., J.B., and D.F.; methodology, D.K., D.T., M.K., S.M., K.B., J.B., Y.J., J.I., and J.Y.; formal analysis, D.T., M.K., K.B., J.B., A.F., and R.P.; data curation, D.T., M.K., A.F., R.P., and K.B.; data verification, D.T. and M.K.; writing—original draft preparation, D.K., D.T., M.K., K.B., and J.B.; writing—review and editing, D.K., D.T., M.K., K.B., J.B., A.F., S.M., R.P., R.M., J.Y., and D.F.; supervision, D.K., M.K., S.M., R.M., Y.J., J.I., J.Y., and D.F. All authors have read and agreed to the published version of the manuscript. All authors have read and approved the final manuscript and consent to its publication in *The Lancet Regional Health – Americas*. Michael Korvink, representing the academic team, had full access to all underlying data reported in this study and verified its accuracy. Drew Tatum, Kathy Belk, Joseph Beals, Ashley Finke, and Raymond Perigard confirm that they had full access to all study data and accept responsibility for the decision to submit the manuscript for publication.

## Data sharing statement

The de-identified dataset used in this study is subject to ethical, legal, and data disclosure restrictions. While the dataset has been anonymized, it contains potentially sensitive patient information that could be indirectly identifiable. These restrictions are in place to comply with privacy regulations (e.g., HIPAA). Additionally, the data is owned by Premier, Inc. and is governed by their Institutional Review Board (IRB) or Research Ethics Committee (REC).

The dataset, as disclosed in the manuscript, includes aggregated data from at least five different healthcare facilities or health systems to ensure privacy and prevent identification of individuals. However, these confidentiality safeguards cannot be maintained if patient-level data is shared, as such disclosure could lead to potential re-identification, especially when the number of facilities represented is limited. Being a commercially available database, access to the PHD is associated with a financial charge.

Requests for access to the de-identified dataset may be submitted to the following data access committee or institutional body:

Michael Korvink. Email: michael_korvink@premierinc.com. Phone: 1 (704) 816–5312.

## Declaration of interests

Dr. Martin reports having received consulting fees from Clinical Computer Systems and American Heart Association, lecturing to interprofessional groups, serving as an expert witness for both defense and plaintiffs, and serving as a member of the AHRQ Maternal Health Indicators Expert Work Group. Dr. Jiang reports receiving consulting fees for statistical review of manuscript. All other authors declare no competing interests.

## References

[1] OECD (2024). https://www.oecd.org/en/data/datasets/oecd-health-statistics.html.

[2] CDC (2024). https://www.cdc.gov/nchs/nvss/vsrr/provisional-maternal-deaths-rates.htm.

[3] Kilday D., Tatum D., Korvink M. (2025). Mortality-weighted severe maternal morbidity: a novel approach to assessing maternal health outcomes. BMC Pregnancy Childbirth.

[4] Fink D.A., Kilday D., Cao Z. (2023). Trends in maternal mortality and severe maternal morbidity during delivery-related hospitalizations in the United States, 2008 to 2021. JAMA Netw Open.

[5] Geller S.E., Koch A.R., Garland C.E., MacDonald E.J., Storey F., Lawton B. (2018). A global view of severe maternal morbidity: moving beyond maternal mortality. Reprod Health.

[6] CDC (2024). Severe Maternal Morbidity.

[7] Office of the Surgeon General (OSG) (2020). http://www.ncbi.nlm.nih.gov/books/NBK568220/.

[8] Brown C.C., Adams C.E., George K.E., Moore J.E. (2020). Associations between comorbidities and severe maternal morbidity. Obstet Gynecol.

[9] Beeson J.G., Homer C.S.E., Morgan C., Menendez C. (2018). Multiple morbidities in pregnancy: time for research, innovation, and action. PLoS Med.

[10] Korst L.M., Gregory K.D., Nicholas L.A. (2021). A scoping review of severe maternal morbidity: describing risk factors and methodological approaches to inform population-based surveillance. Matern Health Neonatol Perinatol.

[11] Leonard S.A., Main E.K., Lyell D.J. (2022). Obstetric comorbidity scores and disparities in severe maternal morbidity across marginalized groups. Am J Obstet Gynecol MFM.

[12] Aoyama K., D’Souza R., Inada E., Lapinsky S.E., Fowler R.A. (2017). Measurement properties of comorbidity indices in maternal health research: a systematic review. BMC Pregnancy Childbirth.

[13] Metcalfe A., Lix L.M., Johnson J.A. (2015). Validation of an obstetric comorbidity index in an external population. BJOG Int J Obstet Gynaecol.

[14] Ruppel H., Liu V.X., Kipnis P. (2021). Development and validation of an obstetric comorbidity risk score for clinical use. Womens Health Rep.

[15] Bateman B.T., Mhyre J.M., Hernandez-Diaz S. (2013). Development of a comorbidity index for use in obstetric patients. Obstet Gynecol.

[16] Easter S.R., Bateman B.T., Sweeney V.H. (2019). A comorbidity-based screening tool to predict severe maternal morbidity at the time of delivery. Am J Obstet Gynecol.

[17] Main E.K., Leonard S.A., Menard M.K. (2020). Association of maternal comorbidity with severe maternal morbidity: a cohort study of California mothers delivering between 1997 and 2014. Ann Intern Med.

[18] Du R., Ali M.M., Sung Y.S. (2023). Maternal comorbidity index and severe maternal morbidity among medicaid covered pregnant women in a US Southern rural state. J Matern-Fetal Neonatal Med.

[19] Murugappan G., Alvero R.J., Lyell D.J., Khandelwal A., Leonard S.A. (2021). Development and validation of a risk prediction index for severe maternal morbidity based on preconception comorbidities among infertile patients. Fertil Steril.

[20] Leonard S.A., Kennedy C.J., Carmichael S.L., Lyell D.J., Main E.K. (2020). An expanded obstetric comorbidity scoring system for predicting severe maternal morbidity. Obstet Gynecol.

[21] Kern-Goldberger A.R., Howell E.A., Srinivas S.K., Levine L.D. (2023). What we talk about when we talk about severe maternal morbidity: a call to action to critically review severe maternal morbidity as an outcome measure. Am J Obstet Gynecol MFM.

[22] Kuklina E.V., Ewing A.C., Satten G.A. (2023). Ranked severe maternal morbidity index for population-level surveillance at delivery hospitalization based on hospital discharge data. PLoS One.

[23] Premier Applied Sciences (2024). https://offers.premierinc.com/Premier-Healthcare-Database-Download.html.

[24] Agency for Healthcare Research and Quality (2025). https://hcup-us.ahrq.gov/toolssoftware/ccsr/ccs_refined.jsp.

[25] CDC (2024). Identifying Severe Maternal Morbidity (SMM).

[26] Sullivan L.M., Massaro J.M., D’Agostino R.B. (2004). Presentation of multivariate data for clinical use: the Framingham Study risk score functions. Stat Med.

[27] van Walraven C., Austin P.C., Jennings A., Quan H., Forster A.J. (2009). A modification of the Elixhauser comorbidity measures into a point system for hospital death using administrative data. Med Care.

[28] Efron B., Tibshirani R. (1994). https://www.taylorfrancis.com/books/mono/10.1201/9780429246593/introduction-bootstrap-bradley-efron-tibshirani.

[29] Elkan C. (2001). Proc Seventeenth Int Conf Artif Intell 4-10 August 2001 Seattle.

[30] R Core Team (2024). https://www.R-project.org/.

[31] Venables W.N., Ripley B.D. (2002). https://www.stats.ox.ac.uk/pub/MASS4.

[32] Singh S., Farber M.K., Bateman B.T. (2022). Obstetric comorbidity index and the odds of general vs. neuraxial anesthesia in women undergoing cesarean delivery: a retrospective cohort study. Int J Obstet Anesth.

[33] Chaalan F., Minisha F., Zaidi Z. (2024). Validation of a modified obstetric comorbidity index for prediction of postpartum adverse events including fetal morbidity - a retrospective cohort study from Qatar. BMC Pregnancy Childbirth.

[34] McCarter A.R., Theiler R.N., Branda M.E., Smith R.M., Sharpe E.E., Torbenson V.E. (2024). The obstetrics comorbidity index as a predictive tool for risk-appropriate maternal care. BMC Pregnancy Childbirth.

[35] Kern-Goldberger A.R., Srinivas S.K., Putt M., Harhay M., Levine L.D. (2023). Implementation of an obstetric co-morbidity scoring system during delivery admissions to reduce maternal morbidity. Am J Obstet Gynecol.

[36] Agency for Healthcare Research and Quality (2025). https://qualityindicators.ahrq.gov/Downloads/Resources/Publications/2025/Empirical_Methods_2025.pdf.

[37] Deneux-Tharaux C., Bouvier-Colle M.H. (2017). 585: severe acute maternal morbidity in France: the epimoms population-based study. Am J Obstet Gynecol.

