# Supplementary material for: A novel maternal prenatal risk index to predict mortality-weighted severe maternal morbidity at hospitalization: a retrospective cohort study
Source: Lancet Reg Health Am. 2026 Apr 24;59:101481. doi: 10.1016/j.lana.2026.101481 (PMC13127213; doi:10.1016/j.lana.2026.101481)

Supplemental Table 1: CDC Abortive Outcome and Birth Volume ICD-10 Definition

**Metric**	**Inclusion/ Exclusion**	**Diagnosis/ Procedure**	**ICD-10 Codes**
Birth Volume	Inclusion	Diagnosis	O75.82, O80, O82, Z37.0, Z37.1, Z37.2, Z37.3, Z37.4, Z37.50, Z37.51, Z37.52, Z37.53, Z37.54, Z37.59, Z37.60, Z37.61, Z37.62, Z37.63, Z37.64, Z37.69, Z37.7, Z37.9
Birth Volume	Inclusion	Procedure	10D00Z0, 10D00Z1, 10D00Z2, 10D07Z3, 10D07Z4, 10D07Z5, 10D07Z6, 10D07Z7, 10D07Z8, 10E0XZZ
Abortive Outcome	Exclusion	Diagnosis	O00.0, O00.1, O00.2, O00.8, O00.9, O01.0, O01.1, O01.9, O02.0, O02.1, O02.81, O02.89, O02.9, O03.0, O03.1, O03.2, O03.30, O03.31, O03.32, O03.33, O03.34, O03.35, O03.36, O03.37, O03.38, O03.39, O03.4, O03.5, O03.6, O03.7, O03.80, O03.81, O03.82, O03.83, O03.84, O03.85, O03.86, O03.87, O03.88, O03.89, O03.9, O04.5, O04.6, O04.7, O04.80, O04.81, O04.82, O04.83, O04.84, O04.85, O04.86, O04.87, O04.88, O04.89, O07.0, O07.1, O07.2, O07.30, O07.31, O07.32, O07.33, O07.34, O07.35, O07.36, O07.37, O07.38, O07.39, O07.4, O08.0, O08.1, O08.2, O08.3, O08.4, O08.5, O08.6, O08.7, O08.81, O08.82, O08.83, O08.89, O08.9
Abortive Outcome	Exclusion	Procedure	10A00ZZ, 10A03ZZ, 10A04ZZ, 10A07Z6, 10A07ZW, 10A07ZX, 10A07ZZ, 10A08ZZ

Confidential

Supplemental Table 2. m-PRI OCSS Modified Conditions Rationale

OCSS Condition	Updated Condition(s)	Rationale
• Anemia, preexisting	• Non-sickle cell anemia<br>• Sickle cell trait/anemia	To isolate differences between sickle cell and non-sickle cell anemia given the differences in severity and management/treatment of anemia throughout gestation. Incorporates the same ICD-10 codes as OCSS condition but broken into mutually exclusive conditions based on coding.
• Cardiac disease, preexisting	• Cardiac disease, acquired<br>• Cardiac disease, congenital	To isolate differences between developed and congenital cardiac conditions given their distinct etiologies and clinical implications. Incorporates the same ICD-10 codes as OCSS condition but broken into mutually exclusive conditions based on coding.
• Delivery BMI > 40	• Obesity	ICD-10 coding guidelines indicate BMI codes should not be assigned during the pregnancy.[1] A set of E66, O26.0, and O99.21 obesity or excessive weight gain codes were used as a proxy for maternal obesity.
• Major mental health disorder	• Mental Health Disorder	Included additional severe self-harm R45.851 and R45.88 codes.
• Placenta accreta spectrum	• Placenta accreta spectrum	Includes additional O43.2 first, second, and unspecified trimester codes to capture early pregnancy risk. Additionally, this broader definition captures conditions that occur during a second or unspecified trimester live birth.
• Placenta previa, complete or partial	• Placenta previa, complete or partial	Includes additional O44 first, second, and unspecified trimester codes to capture early pregnancy risk. Additionally, this broader definition captures conditions that occur during a second or unspecified trimester live birth.

Confidential

• Preexisting diabetes mellitus	• Type I preexisting diabetes mellitus<br>• Type II/Unspecified preexisting diabetes mellitus	To isolate differences between type 1 and type 2 diabetes mellitus due to their varied pathophysiology and treatment. All ICD-10 codes as OCSS, except for Z79.4, were broken into mutually exclusive conditions based on coding. Z79.4 was excluded due to its ambiguity of type I or type II diabetes, and it requires a primary E code that is already part of the type I or type II/unspecified diabetes mellitus definitions.[1]
• Preterm birth (< 37 weeks)	• Preterm birth (< 37 weeks)	Includes additional O60.1 codes indicating preterm labor with preterm delivery.
• Previous cesarean birth	• Previous cesarean birth	Includes additional O34.22 code related to maternal care for a cesarean scar defect indicating a prior cesarean delivery.
• Substance use disorder	• Substance use disorder	Includes additional Z72.0 tobacco use and O99.33 smoking complicating the pregnancy codes.
• Twin/multiple pregnancy	• Multiple gestation	Same ICD-10 codes, but renamed for clarity since a twin pregnancy is a type of multiple pregnancy

Confidential

1. ICD-10-CM Guidelines FY25 October 1 2024. Accessed June 24, 2025. https://www.cms.gov/files/document/fy-2025-icd-10-cm-coding-guidelines.pdf

Supplemental Table 3. m-PRI ICD-10 Condition Definitions

Condition	Codes
**Acquired Cardiac Disease**	I05-I09, I11-I13, I20, I25, I27.8, I31-I32, I34-I39, I44-I45, I47-I48, I49.1-I49.5, I49.8-I49.9, I50.22, I50.32, I50.42, I50.812, O10.1, O10.3, O99.41-O99.42
**Assisted Reproductive Technology**	O09.81
**Asthma (Acute or Moderate/Severe)**	J45.21-J45.22, J45.31-J45.32, J45.4-J45.5, J45.901-J45.902, O99.5
**Bariatric Surgery**	O99.84, Z98.84
**Bleeding Disorder, Preexisting**	D66-D67, D68.0-D68.6, D69
**COVID-19 Infection**	U07.1
**Cancer**	C00-C26, C30-C34, C37-C41, C43-C49, C4A, C50-C58, C60-C79, C7A, C7B, C80-C86, C88, C90-C96, D00-D07, D09-D24, D26-D39, D3A, D40-D49, D61.810, D63.0, D64.81, D70.1, E88.3, G13.1, G73.1, G89.3, H47.42, H47.52, H47.63, J91.0, K12.31, K31.7, K63.5, N52.36, R18.0, R53.0, T80.810A, T80.810D
**Chronic Hypertension**	I10, O10.0, O11
**Chronic Renal Disease**	I12-I13, N03-N05, N07-N08, N11.1, N11.8-N11.9, N18, N25, N26.9, O10.2-O10.3, O26.83
**Congenital Cardiac Disease**	Q20-Q24
**Connective Tissue or Autoimmune Disease**	M30-M36
**Endocrine, Type I Preexisting Diabetes**	E10, O24.0
**Endocrine, Type II Preexisting or Unspecified Diabetes**	E08-E09, E11, E13, O24.1, O24.3, O24.8-O24.9
**Gastrointestinal Disease**	K50-K52, K70-K77, K80-K83, K85-K87, K94-K95, O26.6
**Gestational Diabetes Mellitus**	O24.4
**HIV/AIDS**	B20, O98.7
**Hepatitis Infection**	B15-B19, B25.1, B26.81, B58.1, O98.4
**Immunity Disorders**	D80-D84, D86, D89
**Infection of Amniotic Sac and Membranes**	O41.10, O41.12
**Infection, STI**	A49.3, A50-A60, A63-A65, A74, B00, B37.3, B85.3, B97.7, J16.0, N70-N77, O98.1-O98.3, R85.81-R85.82, R87.81-R87.82
**Mental Health Disorder**	F06, F20-F25, F28-F34, F39, F40.0, F41, F43, F53, F60, R45.851, R45.88
**Multiple Gestation**	O30-O31, O63.2, Z37.2-Z37.7
**Neurologic Disorders**	A32.12, A36.83, A39.81, A42.82, A81.1, A83-A86, A92.2, B01.11, B02.0, B02.21-B02.23, B05.0, B06.01, B10.0, B26.2, B26.84, B40.81, B57.42, B58.2, B60.11, F01-F05, F07.8, F48.2, F51, G04.0, G04.2-G04.3, G04.8-G04.9, G05, G08, G10-G12, G13.0, G13.2, G13.8, G21.0, G21.2-G21.4, G21.8-G21.9, G23, G24.1-G24.5, G24.8-G25.0, G25.2-G25.3, G25.5, G25.69, G25.8-G25.9, G26, G30, G31.0-G31.1, G31.8-G31.9, G32, G35-G37, G47, G50-G61, G62.1, G62.8-G62.9, G63-G64, G71.0, G71.11-G71.13, G71.19, G71.2-G71.3, G71.8-G71.9, G72.1, G72.3-G72.4, G72.8-G72.9, G73.3, G73.7, G90.0-

Confidential

Condition	Codes
	G90.1, G90.3-G90.4, G90.8-G90.9, G90.A, G90.B, G91-G92, G93.0, G93.2-G93.3, G93.40-G93.44, G93.5-G93.9, G94-G96, G98.8, G99, R06.3, R56
**Neuromuscular Disease**	G40, G70
**Non-Sexually Transmitted Infections**	A02.25, A06.7-A06.8, A08.4, A36.85, A65-A68, A69.1, A69.20, A69.22, A69.29, A69.8-A69.9, A75, A77, A79, A90-A91, A92.0-A92.1, A92.3-A92.5, A92.8-A92.9, A93-A96, A98-A99, B01.12, B01.89, B01.9, B02.24, B02.29, B02.7-B02.9, B03-B04, B05.4, B05.89, B05.9, B06.00, B06.09, B06.89, B06.9, B07, B08.0-B08.3, B08.6-B08.8, B09, B10.8, B25.8-B25.9, B26.89, B26.9, B27, B33.0-B33.1, B33.3-B33.4, B33.8, B34, B37.41, B37.49, B37.9, B50-B56, B57.0-B57.2, B57.30, B57.39-B57.40, B57.49, B57.5, B58.89, B58.9, B60.0, B60.10, B60.13, B60.19, B60.2, B60.8, B64, B65.0-B65.2, B65.8-B65.9, B66, B67.32, B67.39, B67.4, B67.6-B67.9, B68, B69.0, B69.89, B69.9, B70-B76, B77.0, B77.89, B77.9-B78.0, B78.7, B78.9, B79-B83, B85.0-B85.2, B85.4, B86-B89, B95.1, B95.62, B96.1, B96.20, B97.0-B97.2, B97.30-B97.34, B97.39, B97.4-B97.6, B97.8, B99, G02, J09-J11, J12.82, J12.89, J15.3, N10, N11.0, N12, N13.6, N15.1, N30, N34, N39.0, O23, O26.4, O75.3, O86.00-O86.03, O86.09, O86.1-O86.2, O86.4, O86.89, O91, O98.0, O98.5-O98.6, O98.8-O98.9, O99.82, T80.211A, T81.4XXA, Z22.330
**Non-Sickle Cell Anemia**	D50-D53, D55-D56, D58-D59, D64.9, O99.01-O99.02
**Obesity**	E66, O26.0, O99.21
**Oligohydramnios**	O41.0
**Other Endocrine Conditions**	A39.1, B67.31, E00-E02, E03.0-E03.1, E03.3-E03.5, E03.8-E03.9, E04, E06.0-E06.3, E06.5, E06.9, E07, E15, E16.1-E16.4, E16.8-E16.9, E20-E22, E23.0, E23.2-E23.3, E23.6-E23.7, E24.0-E24.1, E24.3-E24.4, E24.8-E24.9, E25-E26, E27.0-E27.2, E27.4-E27.5, E27.8-E28.2, E28.39, E28.8-E28.9, E30-E32, E34-E35, E65, E67, E70-E80, E83, E85, E88.0-E88.2, E88.4, E88.8-E88.9, E88.A, O90.5
**Placenta Accreta Spectrum**	O43.2
**Placenta Previa, Complete or Partial**	O44.0-O44.3
**Placental Abruption**	O45.001, O45.011, O45.021, O45.091, O45.8-O45.9
**Polyhydramnios**	O40
**Preeclampsia With Severe Features**	O11, O14.1-O14.2
**Preeclampsia Without Severe Features or Gestational Hypertension**	O13, O14.0, O14.9
**Preterm Birth (Less Than 37 Wk)**	O60.1, Z3A.2, Z3A.30, Z3A.31, Z3A.32, Z3A.33, Z3A.34, Z3A.35, Z3A.36
**Previous Cesarean Birth**	O34.21-O34.22, O66.41
**Pulmonary Hypertension**	I27.0, I27.2

Confidential

Condition	Codes
**Renal, Other**	A36.84, B26.83, B58.83, N00-N01, N02.B, N14.0, N14.4, N15.0, N15.8-N15.9, N16, Z99.2
**Ruptured Membranes, Other**	O42.00, O42.02, O42.10, O42.12, O42.90, O42.92, O75.5
**Ruptured Membranes, PPROM**	O42.01, O42.11, O42.91
**Sickle Cell Trait/Anemia**	D57.1, D57.20, D57.3, D57.40, D57.80
**Substance Use Disorder**	F10-F19, F55, O99.31-O99.33, Z72.0
**Thyrotoxicosis**	E05
**Uterine Fibroids**	D25, O34.1

Confidential

Supplemental Table 4. Validation Set Confusion Matrix Metrics by Ordinal Class

	m-PRI			OCSS			Percentage Difference		
Class	Precision	Recall	F1	Precision	Recall	F1	Precision Difference	Recall Difference	F1 Difference
SI Level 1	0.997	0.792	0.883	0.996	0.806	0.891	0.1%	-1.8%	-1.0%
SI Level 2	0.012	0.307	0.023	0.010	0.202	0.019	20.3%	52.1%	21.5%
SI Level 3	0.013	0.185	0.025	0.008	0.167	0.015	73.4%	10.7%	69.2%
SI Level 4	0.031	0.490	0.058	0.017	0.508	0.034	77.2%	-3.4%	72.4%

Confidential

Supplemental Table 5. Patient and Hospital Characteristic Distributions between Derivation and Validation Datasets

Characteristic	Overall N (%)	Derivation n (%)	Validation n (%)	SMD
Total N	7,174,412	6,341,166	833,246	
Maternal Age				0.083
Age (mean/SD)	29.06 (5.78)	29.01 (5.78)	29.49 (5.77)	
Race				0.082
American Indian/Alaska Native	61,080 (0.9)	53,889 (0.8)	7,191 (0.9)	
Asian	334,958 (4.7)	293,572 (4.6)	41,386 (5.0)	
Black	1,074,767 (15.0)	947,557 (14.9)	127,210 (15.3)	
Other	746,533 (10.4)	667,906 (10.5)	78,627 (9.4)	
Pacific Islander	71,904 (1.0)	65,611 (1.0)	6,293 (0.8)	
Unknown	345,024 (4.8)	293,794 (4.6)	51,230 (6.1)	
White	4,540,146 (63.3)	4,018,837 (63.4)	521,309 (62.6)	
Ethnicity				0.285
Hispanic or Latino	1,280,737 (17.9)	1,096,941 (17.3)	183,796 (22.1)	
Not Hispanic or Latino	4,856,503 (67.7)	4,264,289 (67.2)	592,214 (71.1)	
Unknown	1,037,172 (14.5)	979,936 (15.5)	57,236 (6.9)	
Payer				0.043
Charity or Indigent	8,727 (0.1)	7,916 (0.1)	811 (0.1)	
Commercial insurance	3,726,993 (51.9)	3,282,846 (51.8)	444,147 (53.3)	
Medicaid	3,027,548 (42.2)	2,686,498 (42.4)	341,050 (40.9)	
Medicare	40,336 (0.6)	37,092 (0.6)	3,244 (0.4)	
Other/Unknown	370,808 (5.2)	326,814 (5.2)	43,994 (5.3)	
Admission Type				0.116
Elective	3,853,433 (53.7)	3,402,209 (53.7)	451,224 (54.2)	
Unknown	292,694 (4.1)	273,798 (4.3)	18,896 (2.3)	
Trauma/Emergency/Urgent	3,028,285 (42.2)	2,665,159 (42.0)	363,126 (43.6)	
Admission Source				0.094
Unknown/Other	67,266 (0.9)	56,908 (0.9)	10,358 (1.2)	
Clinic	1,310,680 (18.3)	1,182,955 (18.7)	127,725 (15.3)	
Non healthcare facility	5,700,086 (79.5)	5,016,798 (79.1)	683,288 (82.0)	
Transfer from other healthcare facility	96,380 (1.3)	84,505 (1.3)	11,875 (1.4)	
Discharge Status				0.020
Expired	433 (<0.1)	388 (<0.1)	45 (<0.1)	
Home/Home Health	7,147,474 (99.6)	6,317,847 (99.6)	829,627 (99.6)	
Other/Unknown	11,135 (0.2)	9,231 (0.1)	1,904 (0.2)	
Transfer	15,370 (0.2)	13,700 (0.2)	1,670 (0.2)	
Delivery Type				0.007
Cesarean	2,329,900 (32.5)	2,056,849 (32.4)	273,051 (32.8)	
Vaginal	4,844,512 (67.5)	4,284,317 (67.6)	560,195 (67.2)	

Hospital Bed Size				0.049
0-299	2,597,817 (36.2)	2,280,217 (36.0)	317,600 (38.1)	
300-499	2,087,717 (29.1)	1,858,072 (29.3)	229,645 (27.6)	
500+	2,488,878 (34.7)	2,202,877 (34.7)	286,001 (34.3)	
COTH Status				0.012
Non-Teaching Hospital	5,261,196 (73.3)	4,654,026 (73.4)	607,170 (72.9)	
Teaching Hospital	1,913,216 (26.7)	1,687,140 (26.6)	226,076 (27.1)	
HHS Region				0.156
Region 1 Boston	205,650 (2.9)	172,051 (2.7)	33,599 (4.0)	
Region 2 New York	642,181 (9.0)	572,329 (9.0)	69,852 (8.4)	
Region 3 Philadelphia	783,521 (10.9)	707,580 (11.2)	75,941 (9.1)	
Region 4 Atlanta	1,926,909 (26.9)	1,673,117 (26.4)	253,792 (30.5)	
Region 5 Chicago	1,108,542 (15.5)	988,941 (15.6)	119,601 (14.4)	
Region 6 Dallas	865,602 (12.1)	774,884 (12.2)	90,718 (10.9)	
Region 7 Kansas City	370,655 (5.2)	323,907 (5.1)	46,748 (5.6)	
Region 8 Denver	158,351 (2.2)	136,710 (2.2)	21,641 (2.6)	
Region 9 San Francisco	836,924 (11.7)	738,498 (11.6)	98,426 (11.8)	
Region 10 Seattle	276,077 (3.8)	253,149 (4.0)	22,928 (2.8)	
Level 3 NICU				0.009
No	3,716,112 (53.8)	3,290,753 (53.8)	425,359 (53.9)	
Yes	3,167,251 (45.9)	2,806,072 (45.9)	361,179 (45.7)	
Unknown	22,351 (0.3)	19,423 (0.3)	2,928 (0.4)	
Hospital Annual Birth Volume				0.043
1 to 99	5,933 (0.1)	5,318 (0.1)	615 (0.1)	
100 to 499	369,047 (5.1)	330,786 (5.2)	38,261 (4.6)	
500 to 999	823,239 (11.5)	726,700 (11.5)	96,539 (11.6)	
1,000 to 1,999	1,651,585 (23.0)	1,468,028 (23.2)	183,557 (22.0)	
2,000 to 3,999	2,914,962 (40.6)	2,568,403 (40.5)	346,559 (41.6)	
4,000+	1,409,646 (19.6)	1,241,931 (19.6)	167,715 (20.1)	
Population Served				0.041
Rural	700,208 (9.8)	627,712 (9.9)	72,496 (8.7)	
Urban	6,474,204 (90.2)	5,713,454 (90.1)	760,750 (91.3)	

Supplemental Table 6. PR-AUC Estimates Across Sensitivity Analysis Model Specification

Condition Set	PR-AUC Estimate	Model and Outcome	Incremental Difference	Model Specification Notes
OCSS (unaltered definitions)	0.173	TMLE (Binary SMM as outcome)		Weights based on published results, applied to Premier data.
OCSS (unaltered definitions)	0.194	Ordinal Regression (Weighted SMM as outcome)	0.021	HIV/AIDS, connective tissue or autoimmune disease, gestational diabetes, and thyrotoxicosis were assigned a weight of zero. Delivery BMI ≥ 40 and previous cesarean birth received non-zero weights. The three largest weights were placenta accreta spectrum, chronic renal disease, and preexisting cardiac disease.
OCSS (modified definitions)	0.206	Ordinal Regression (Weighted SMM as outcome)	0.012	All prior conditions and associated modification were retained, except congenital cardiac disease (acquired cardiac disease retained), type II or unspecified preexisting diabetes (type I retained), and sickle cell anemia or trait (non-sickle cell anemia was retained). The three largest weights were placenta accreta spectrum, chronic renal disease, and acquired cardiac disease. Note: OCSS's preexisting cardiac disease was split into acquired and congenital cardiac disease.
m-PRI (OCSS modified + new conditions)	0.223	Ordinal Regression (Weighted SMM as outcome)	0.017	Type I preexisting diabetes, obesity, and major mental health disorder were assigned a weight of zero. Eight new conditions were given non-zero weights. The largest three weights remained unchanged.

Confidential

Supplemental Figure 1. SMM Composite Distribution

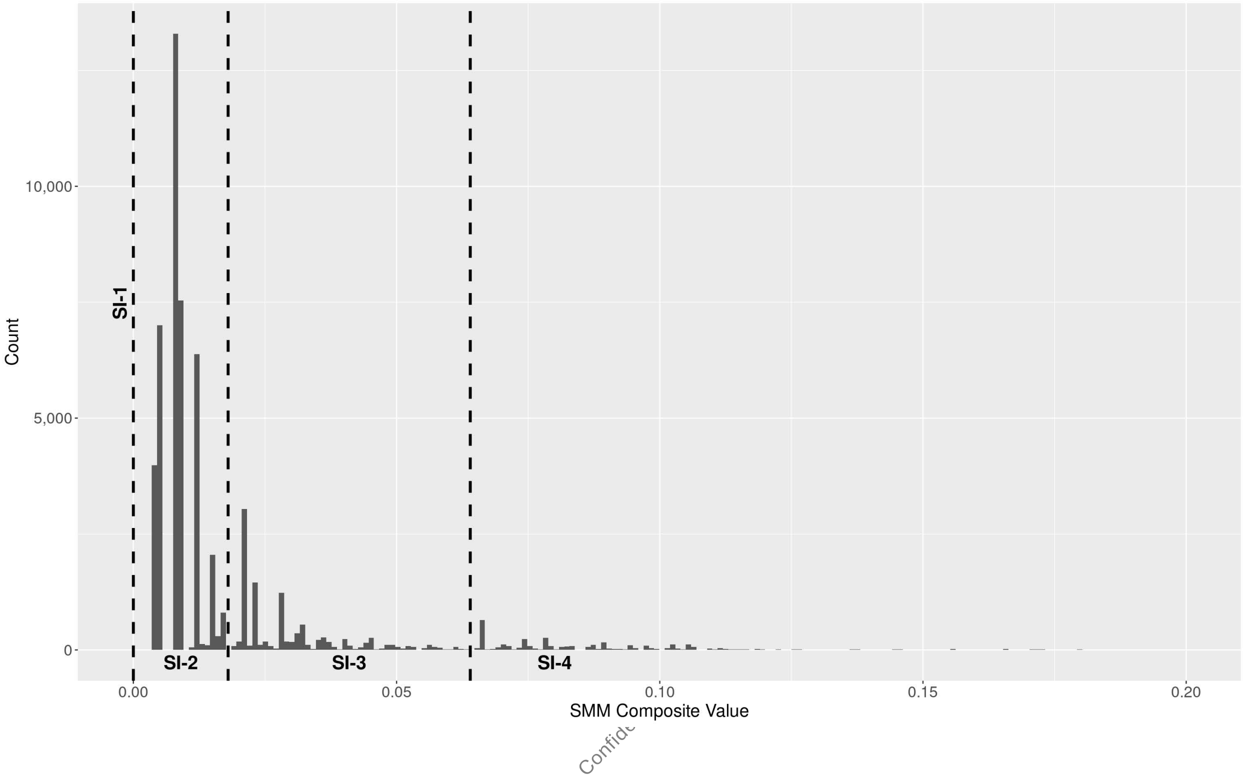

Supplemental Figure 2. m-PRI Confusion Matrix for SMM-Severity Group on Validation Set

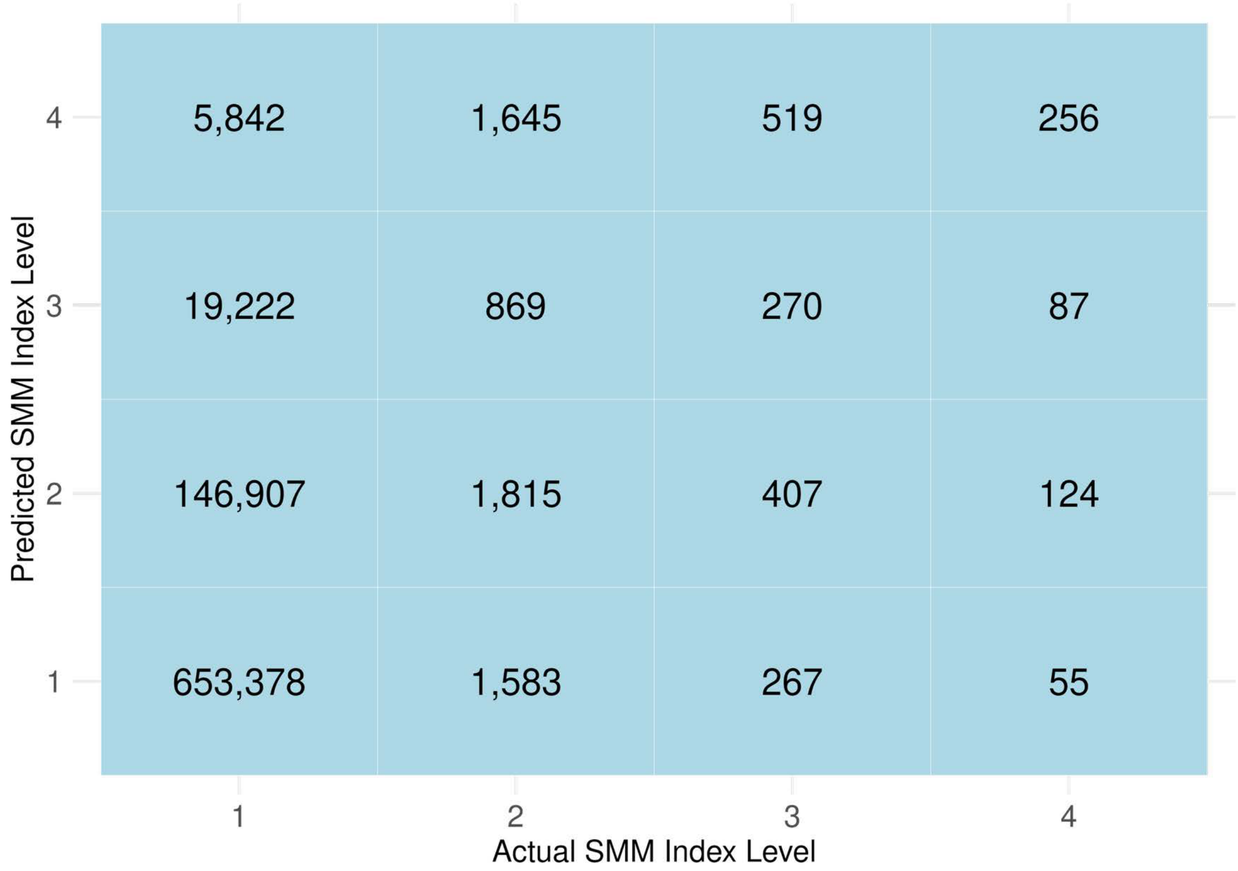

Supplemental Figure 3. OCSS Confusion Matrix for SMM-Severity Group on Validation Set

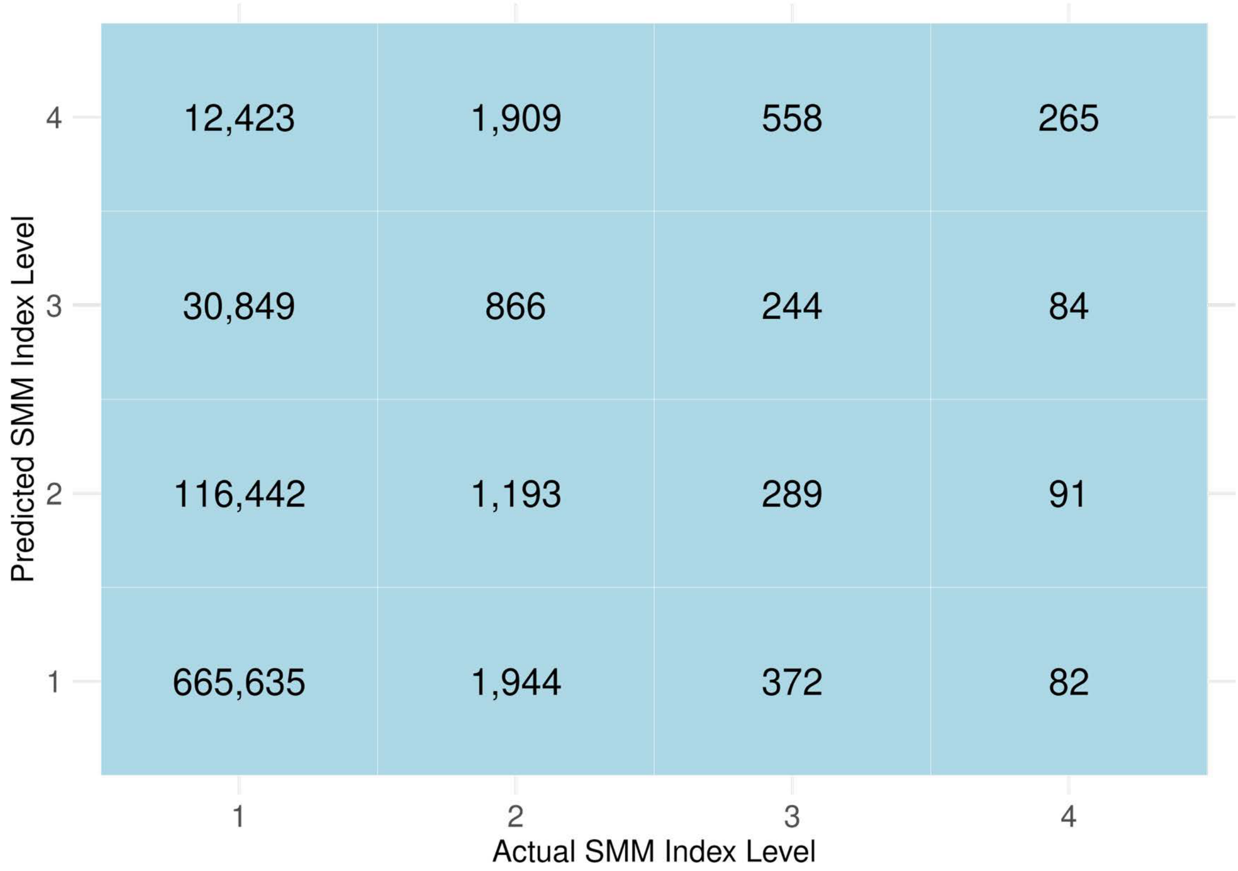

Supplemental Figure 4: Box Plot Illustrating m-PRI Distribution by SMM Severity Levels

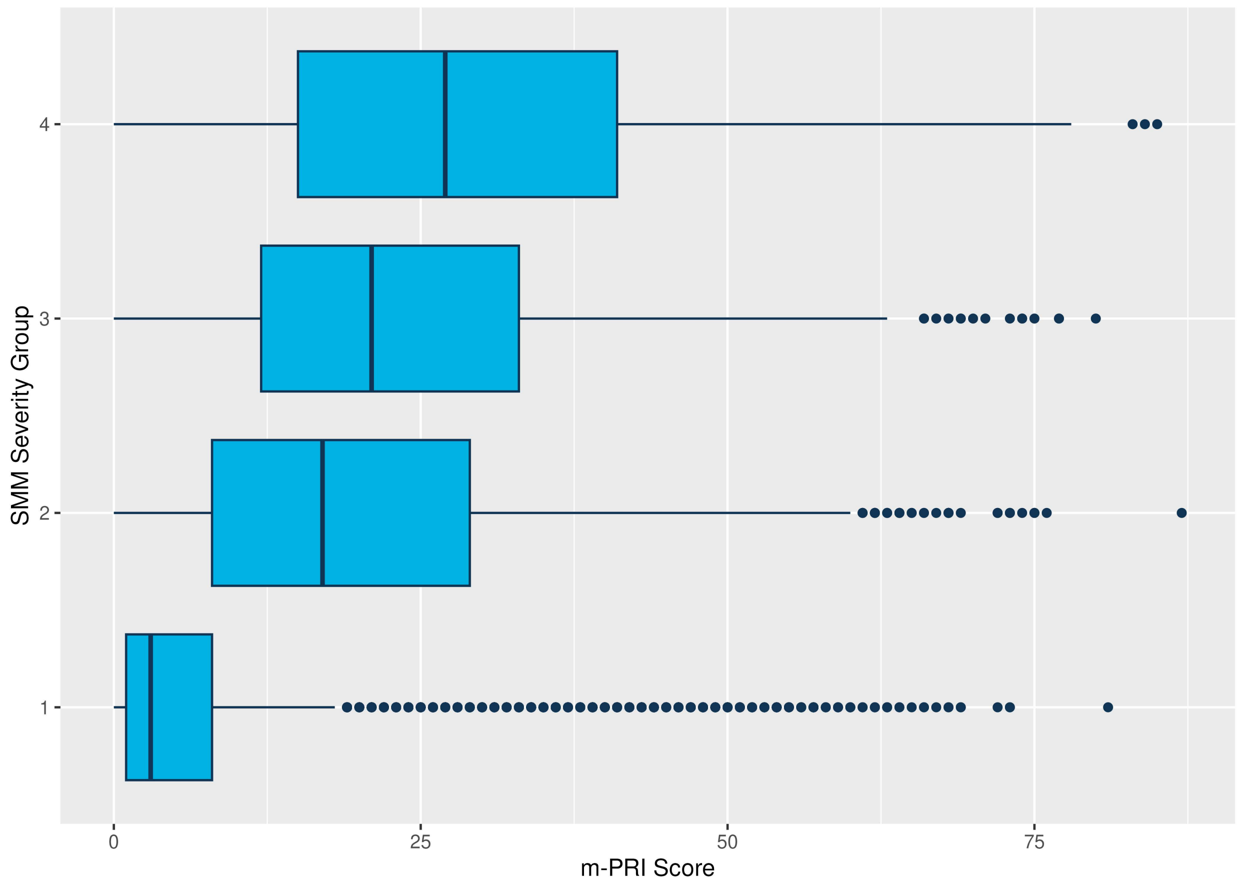

Supplement: Supplemental Figs. S1–S4 and Tables S1–S6 [file mmc1.pdf]
